# The Effects of Host-Feeding on Synovigenic Egg Development in An Endoparasitic Wasp, *Itoplectis naranyae*

**DOI:** 10.1673/031.007.4601

**Published:** 2007-08-28

**Authors:** Takatoshi Ueno, Kanako Ueno

**Affiliations:** Institute of Biological Control, Faculty of Agriculture, Kyushu University, Fukuoka 812-8581, Japan

**Keywords:** reproduction, nutrition, anhydropic eggs, pupal parasitoids, Pimplinae, oogenesis

## Abstract

Many adult parasitoids feed on host insects, a behavior known as host-feeding. Feeding on hosts is essential to maximizing female fecundity, but its contribution to reproduction varies from species to species. The relationship between fecundity and host-feeding was examined in the solitary endoparasitoid wasp *Itoplectis naranyae* Ashmead, (Hymenoptera: Ichneumonidae) to assess the significance of host-feeding in female reproduction. Adult female wasps did not respond to hosts when they were 0–1 days old, but subsequently increased their oviposition and host-feeding activities with increasing female age. While newly emerging females had no mature eggs in their ovary, the number of mature eggs increased rapidly thereafter, a process termed synovigeny. Female wasps were capable of maturing eggs without host-feeding, and this suggested that they produced a certain portion of eggs from nutritional reserves that had been stored during the larval stage. Behavioral observations revealed that *I. naranyae* was a destructive host-feeder as the host was damaged during feeding. Female fecundity was greater in females that had previously fed on hosts than those did not, indicating that host-feeding was involved in egg production. There was a time-delayed relation between host-feeding events and additional egg production; at least 3 days were required to mature eggs from nutrients gained via feeding on hosts. The significance of host-feeding in *I. naranyae* reproduction is discussed in the context of its life history traits.

## Introduction

Insect parasitoids are among one of the most diverse groups of insects, exhibiting a variety of life-history strategies that have fascinated many researchers in evolutionary and behavioral ecology (Gauld and Bolton 1988; Godfray 1994; Hawkins and Sheehan 1994; Quicke 1997). Parasitoids can also play an essential role in controlling insect herbivore populations, which makes this group of insects important for many applied entomologists. Revealing the life-history characteristics of parasitoids is the primary approach to assess their importance and effectiveness as natural enemies against insect pests (Jervis and Kidd 1996; Hawkins and Cornell 1999). Thus, parasitoid life history has been studied from both basic and applied points of view.

Reproductive ecology in parasitoids has been of great interest because it is a major component of their life history as it relates to egg production and female fecundity (Price 1973, 1974; Quicke 1997; Papaj 2000). Nutrition is the single most important factor influencing egg production and female fecundity; the amount of resources available to the female from her own larval and adult feeding largely determines her reproductive potential (Godfray 1994; Quicke 1997; Thompson 1999; Papaj 2000; Rivero et al. 2001).

The influence of larval nutrition (usually measured as host size or wasp size) on fecundity has been widely investigated. In most cases, the fecundity of female parasitoids is correlated with their body size and, hence, with the amount of stored nutrient reserves acquired during the larval stage (e.g. Waage and Godfray 1985; Godfray 1994; Harvey et al. 1994; Visser 1994). Thus, larval nutrition is important in oogenesis.

Adult nutrition is also important in female reproduction. Adult parasitoids after eclosion generally require food to sustain foraging activity and to initiate and maintain oogenesis (Jervis and Kidd 1986; Heimpel and Collier 1996; Jervis et al. 1996; Thompson 1999). Plant materials such as floral and extrafloral nectars, pollens etc. are widely used as adult food (Jervis et al. 1993, 1996; Thompson 1999). In addition, the adults of a number of hymenopteran parasitoid species use their host as food (Jervis and Kidd 1986; Heimpel and Collier 1996; Thompson 1999). In such parasitoids, adult females feed on host body fluids, an action called ‘host-feeding’. Host-feeding is an essential means of obtaining protein resources for producing eggs (e.g., Sandlan 1979; Collier 1995a; Rivero and Casas 1999; Ueno 1999a; Rivero et al. 2001). In some parasitoids, females cannot produce any eggs, or can produce only a small fraction of the maximum number of eggs, when host-feeding is not allowed (Leius 1962; Ueno 1999a). Host-feeding can therefore be essential to female reproduction.

However, not all parasitoids feed on hosts (Jervis and Kidd 1986). Life history characteristics in a given parasitoid species can determine the importance of adult nutrition and host-feeding in a species. Koinobiont larval parasitoids, which carry many small, yolk-deficient eggs at eclosion, are mostly non-host feeders (Jervis and Kidd 1986; Jervis et al. 2001). Parasitoids that produce large, yolk-rich eggs will require relatively large amounts of resources for egg production, and, hence, nutrition acquired during the larval stage may cover only a small proportion of nutrients used for egg production: Adult nutrition and host-feeding would thus be important in such parasitoids.

Parasitoids that produce relatively large, yolk-rich eggs carry a relatively limited number of mature eggs at any one time because the maximum egg load is relatively small and because maturing many costly eggs at any one time could be difficult. In fact, such parasitoids produce mature eggs repeatedly during their lifetime, and many of them gain nutrients for egg production by feeding on hosts frequently during their lifetime. This reproductive mode, in which adult females mature eggs throughout their life, is called ‘synovigeny^’^ (Jervis and Kidd 1986; Thompson 1999; Jervis et al. 2001). Speices that produce large yolk-rich of eggs are generally synovigenic.

Consequently, close relationships between synovigeny, egg morphology (size, yolk, etc.) and host-feeding behavior would arise, and all of these can reflect to what degree adult nutrition contributes to female reproduction (Jervis and Kidd 1986; Jervis et al. 2001). Examining the links between these three reproductive traits is therefore important for the understanding the fundamentals of host-feeding and reproduction in parasitoids. In the current study, we examine reproductive modes, i.e. synovigeny, as well as relationships between host-feeding and fecundity in a parasitoid wasp.

For synovigenic host-feeders, reproductive behavior in the early stage of female life may particularly be important. If females cannot produce any eggs without host food, host-feeding would then precede oviposition (Jervis and Kidd 1986); they use the first series of hosts encountered exclusively for host-feeding. If newly eclosed females can produce some mature eggs without host-feeding, they would oviposit first. The timing of the first host-feeding may be determined by the amount of nutrients that were stored during the larval stage. Thus, investigation of oviposition and host-feeding behavior in the early stage of female life is essential to studying reproductive strategy in parasitoids.

The solitary endoparasitoid wasp *Itoplectis naranyae* Ashmead (Hymenoptera: Ichneumonidae) is a polyphagous species that attacks a variety of lepidopterous pupae and prepupae including *Chilo suppressalis* and *Cnaphalocrocis merdinalis* Guenee, which are important pests in rice paddies (Townes et al. 1965; Yasumatsu and Watanabe 1965; Ueno and Tanaka 1994). Female *I. naranyae* produce relatively large yolk-rich eggs, and have relatively few mature eggs at any one time during oviposition periods (Iwata 1960; Ueno and Tanaka 1994). Production of such large, yolk-rich eggs should be costly for the female wasp, and adult nutrition would then be important for *I. naranyae*. Previous studies have demonstrated that this parasitoid is a host-feeder (Ueno 1998; Ueno and Ueno 2004), like other members of pimpline parasitoids (Sandlan 1979; Ueno 1999a).

Here a series of experiments were done to confirm whether *I. naranyae* is a synovigenic parasitoid. Although our previous study has suggested that female *I. naranyae* eclose with no or few mature eggs (Ueno and Tanaka 1994), the egg production schedule is still unknown. Therefore, we focused on reproductive behavior (oviposition and host-feeding) and egg production during early phases of the adulthood. The presence of eggs in the ovary was also examined because it strongly reflects egg production strategy of parasitoids (Price 1972). To assess relationships between host-feeding and fecundity in *I. naranyae*, we tested whether females that had fed on hosts had greater egg loads than those did not. We discuss the importance of host-feeding in *I. naranyae* reproduction, and its relationships with the life history characteristics.

## Materials and Methods

### Parasitoid and host

A laboratory colony of *I. naranyae* was established using adult parasitoids collected from Tsukuba and Fukuoka City, Japan. Female parasitoids were placed individually in plastic containers (10 cm in diameter, 4.5 cm in height), together with tissue paper saturated with diluted honey. The tissue paper was replaced twice a week thereafter. The containers were kept at 20 ± 1°C.

The colony was maintained on pupae of a laboratory host, *Galleria mellonella* (Linnaeus) (Lepidoptera: Pyralidae). Host cocoons containing fresh pupae were presented to female *I. naranyae* in the plastic containers. Parasitized hosts were removed from the container and held at 20 ± 0.5 °C until wasp eclosion. Newly eclosed wasps were paired and placed in the plastic containers and maintained as mentioned above.

### Host-feeding behavior

Direct behavioral observations were made to investigate the characteristics of host-feeding behavior in *I. naranyae*. For this purpose, females of *I. naranyae* were given a series of hosts at random, and their responses to hosts were directly observed. In some cases, females were carefully observed under a binocular microscope to investigate host-feeding behavior in detail.

### Reproductive behavior in early phases of adult life

Newly eclosed females of *I. naranyae* were individually placed in plastic containers together with a male. Tissue paper saturated with diluted honey was also placed in the container, and was replaced every two days thereafter to provide females with fresh food. Females of *I. naranyae* eclose with no mature eggs and hence are reproductively inactive when they are 0–1 days old. Experiments were therefore conducted with 3 day-old females.

On the third day after female eclosion, females for testing were provided with fresh host cocoons for 2 hours, and were allowed free access to the hosts. Hosts were then removed from the container, and were examined under a binocular microscope to count the oviposition and host-feeding holes left by females. Holes made by the ovipositor were easily detected on the surface of a host pupa. To determine whether the hosts had been fed on note was taken whether hemolymph exuded from hosts. When conspicuous marks of host body fluid exudation were observed on a host, it was classified as a ‘fed’ host. This procedure was repeated for 5 consecutive days, and thus a 5-day experimental period was used for each test female.

After the experimental period, forewing length of test females was measured under a binocular microscope to use as an index of female size. In all, 41 females were used in the experiment, and no test females died during the experimental period.

### Egg production in early phases of adult life

This experiment was carried out to examine egg production patterns during the early stage of female life. Female wasps aged 0, 1, 2, 4, 7, and 10 days old were dissected under a binocular microscope. Changes in the contents of the ovary and ovarioles were observed, and the number of mature and immature eggs a female carried were counted. Eggs that were well formed with a smooth surface were categorized as mature eggs, and others with a rough surface, or attached by nurse cells, as immature eggs. Females were provided with tissue paper saturated with a diluted honey, but were not allowed access to hosts. Eighty-five females were used in this experiment.

### Egg maturation delay

To examine the time required to convert ingested host materials to eggs, the number of eggs that females carried was compared between females that fed on hosts and those had not fed. Naïve females aged 8 days old were allowed to attack 2 hosts for 2 hours. Each female was categorized by whether it had oviposited in hosts or females that had oviposited and host fed. After 2 days, females were dissected to count the number of mature eggs that they carried. Likewise, the number of mature eggs was compared between females that had host fed 3 days previously and those not. In this experiment, 18 and 28 females, respectively, were used for dissection.

### Voucher specimens

Specimens of *I. naranyae* used in the present study were deposited at the Institute of Biological Control, Kyushu University.

### Statistical analyses

Statistical treatments were made with the aid of Statview (SAS Institute 1999) and JMP (SAS Institute 2001). Normality had been checked before parametric procedures were applied. When normality of the data was not satisfied, non-parametric statistical treatments were used. Paired *t* tests, where appropriate, were also used.

## Results

### Host-feeding behavior

Upon feeding on a host, female *I. naranyae* mostly used the ovipositor to make holes in the integument of the host to extract host fluids. After thrusting into a host with the ovipositor, the female moved her ovipositor in a circular way thereby making enlarging the hole in the host that facilitated host fluids exuding from the hole. The female then consumed the fluids. This series of behaviors was repeated. Consequently, hosts that had been fed on were heavily damaged with conspicuous marks of multiple cuticle penetrations. Thus, host-feeding type of *I. naranyae* was categorized as ‘destructive’ (*sensu* Jervis and Kidd, 1986). Females occasionally sipped host fluids exuded from the oviposition hole after oviposition, which was not followed by a typical circular movement of the abdomen. In this case, hosts were not damaged, and, in this sense, this type of feeding could be regarded as non-destructive host-feeding. Only destructive types of host-feeding were analyzed in the following experiments because it was difficult to examine whether hosts had been used for non-destructive host-feeding.

### Reproduction behavior in early phases of adult life

**Figure 1.**
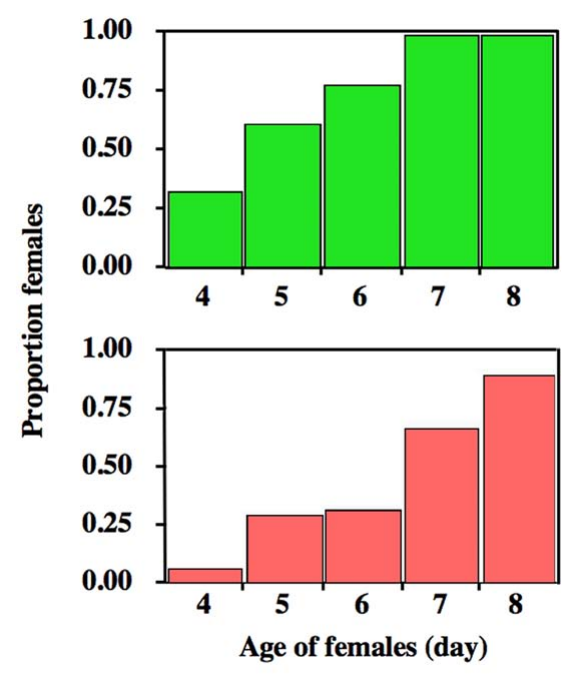
Proportions of females that oviposited (above) and those that host fed (below) in relation to female age. The proportions differed among the age classes (*χ*2 test, P < 0.0001).

Oviposition and host-feeding activity of female *I. naranyae* depended strongly on female age (*χ*^2^ test; N = 42, *χ*^2^ = 71.3, P < 0.0001 for proportion of females oviposited; *χ*2 = 82.6, P < 0.0001 for proportion of females host-fed). The proportion of females that oviposited and those host-fed quickly increased with increasing female age (Figure 1). Less than 25% of females oviposited in hosts when they were 4 days old, but by the 7^th^ day all females had oviposited (Figure 1). The results suggest a 3- to 6-day-pre-ovipositional period in *I. naranyae*.

**Figure 2.**
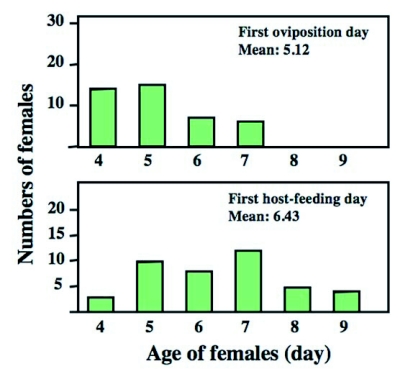
The first day when individual females oviposited (above) and host fed (below) during the early stage of their life. Host-feeding followed by oviposition. Mean days differed significantly between the two types of behavior (Paired t-test, P < 0.0001). Data were shown as means ± SE.

Among 42 test females, 15 females oviposited and host-fed for the first time on the same day. The remaining females started to host feed a few days after the first oviposition. The mean number of days that females oviposited and host-fed for the first time were 5.12 ± 0.16 (± SE) and 6.43 ± 0.22 (± SE), respectively (Figure 2). A paired *t*-test revealed that these differences were significant (*t* = -6.11, P < 0.0001). This result demonstrates that host-feeding was generally followed by oviposition for each female. Thus, females used at least the first several hosts for oviposition purposes only.

**Figure 3.**
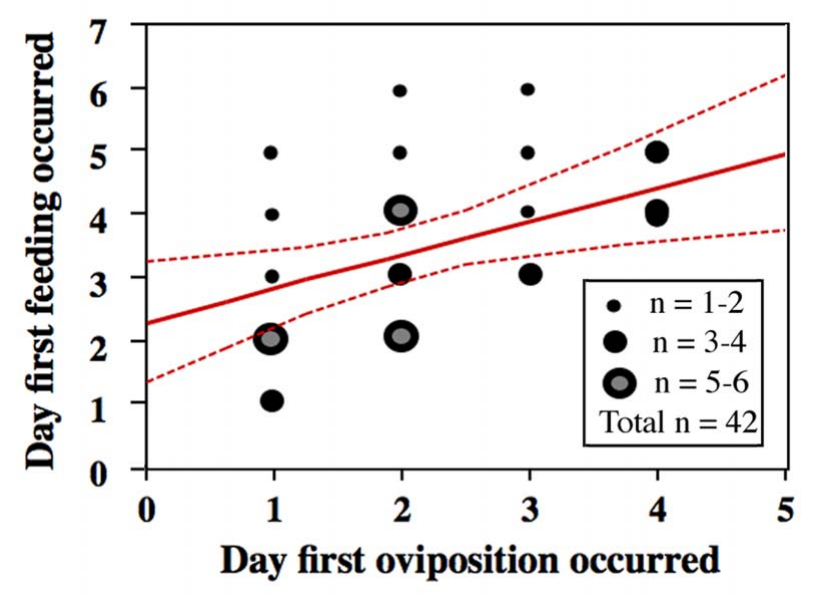
Relationship between the first days of oviposition and host-feeding. Regression was significant (P < 0.01). Females that had oviposited earlier fed on hosts earlier in their lifetime.

**Figure 4.**
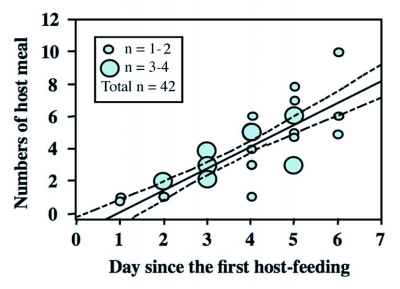
Relationship between days since the first host-feeding and the number of host meals (= feeding events). Regression obtained was significant (P < 0.0001). Females frequently fed on hosts during the early stage of the lifetime.

**Figure 5.**
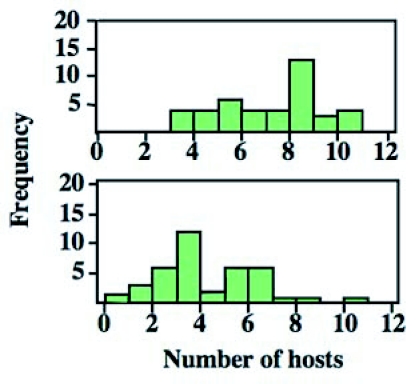
Individual variation in total numbers of hosts that were parasitized (above) and host fed (below) during the experiment.

**Figure 6.**
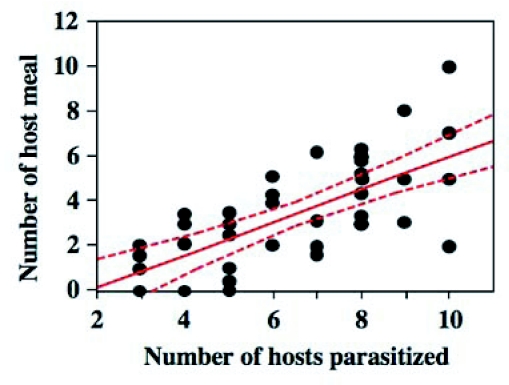
Relationship between the number of hosts parasitized and those fed on (Regression analysis, P < 0.0001). Females that had oviposited in more hosts fed on more hosts.

**Figure 7.**
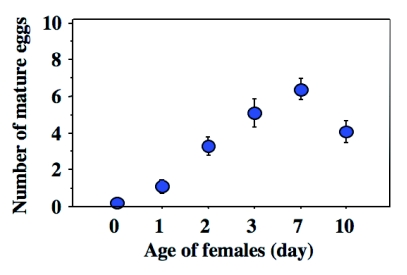
Relationship between female age and the number of mature eggs each female carried in the ovary. Females eclosed with no mature eggs, and the number of eggs increased with increasing female age (Kendall's rank correlation test, P < 0.001). Data were shown as means ± SE.

The results summarized in Figure 1 and 2 showed that there was great variation in the initiation of oviposition and host-feeding among females. Accordingly, the relationship between the first day of oviposition and host-feeding was examined. A positive relationship was detected between the two events (Regression analysis; N = 42, *r*^2^ = 0.16, F = 7.38, P = 0.0097) (Figure 3). Therefore, females that started to oviposit earlier began to feed on hosts earlier.

During the early phase of female life, *I. naranyae* frequently fed on hosts. The number of hosts that had been fed upon by a female increased since the first host-feeding event; there was a positive relationship between the number of hosts fed on and the day since the first host-feeding (Regression analysis; N = 42, *r*^2^ = 0.72, F = 100.7, P < 0.0001) (Figure 4). The present result demonstrates that females continuously use a certain number of hosts for host-feeding during the early stage of female life, probably accumulating nutrients for future egg production.

The total numbers of hosts used for oviposition and host-feeding varied greatly among test females (Figure 5). Accordingly, the relationship between the number of host fed on and that of hosts parasitized was analyzed. There was a strong positive relationship between the number of oviposition and host-feeding events (Regression analysis; N = 42, *r*^2^ = 0.47, t = 5.97, P < 0.0001) (Figure 6). This suggested that the more the female oviposited, the more hosts she fed on, demonstrating a close relationship between oviposition and host-feeding activities in *I. naranyae*.

To assess how many times a female penetrated the cuticle during host-feeding, the numbers of holes made with the ovipositor were compared between oviposited and fed hosts. A Wilcoxon's test showed the numbers of holes were greater in fed hosts than oviposited hosts (mean ± SE; 11.4 ± 1.3 versus 4.06 ± 0.6, *χ*^2^ = 22.7, P < 0.0001). This suggested that they drill into hosts 7–8 times during each host-feeding event.

**Figure 8.**
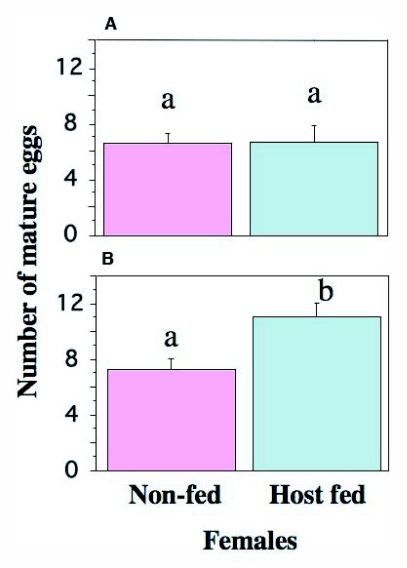
The mean numbers of mature eggs carried by host-fed and non host-fed females, demonstrating egg maturation delay from host-feeding (A, 2 days after host-feeding; B, 3 days after host-feeding). Different letters above bars indicate a significant difference between the groups (ANOVA, P < 0.05). Lines above bars indicate SE.

### Egg production in early phases of adult life

The dissection revealed that a female of *I. naranyae* had 10–24 ovarioles and eclosed with no mature eggs in her ovary (Figure 7). No accumulation of yolk was observed in ovarioles of newly eclosed females. However, oocytes quickly started to develop after adult eclosion, and each of ovarioles contained at least one developing oocyte (i.e. immature egg) or one mature oocyte (mature egg). Accordingly, the number of mature eggs per female increased as females aged (Kendall's rank correlation test; P < 0.0001). Females carried 4–6 mature eggs in the ovary by 5 days after eclosion (Figure 7).

### Egg maturation delay

The dissection revealed that the number of mature eggs did not differ between females that had fed on a host 2 days previously and those not fed (Figure 8a) (mean eggs; 6.6 ±1.1 versus 6.7 ± 0.9; ANOVA, F = 0.003, P = 0.95). However, females that had fed on a host 3 days previously carried more mature eggs than those not fed 3 days previously (Figure 8b) (mean eggs; 11.1 ± 0.9 versus 7.8 ± 1.1; ANOVA, F = 5.48, P = 0.027). Host-feeding therefore allowed female *I. naranyae* to produce additional eggs but there was a time delay between the feeding event and the production of new eggs.

## Discussion

### Synovigeny and life history traits of *I. naranyae*

The present observations indicate that *I. naranyae* is typically synovigenic (Figure 1). Previous research has suggested that ‘synovigeny’ is often linked by a number of reproductive and life history traits of parasitoids though there are many exceptions (Jervis and Kidd 1986; Jervis et al. 2001). These traits include:

- production of relatively large, yolk-rich eggs
- relatively low fecundity at any one time
- relatively long life span
- low oviposition activity in the earliest stage of female life and the subsequent increase in oviposition activity, which can remain stable thereafter
- host-feeding behavior.

*I. naranyae* has all of these traits. First, *I. naranyae* produced relatively large, yolk rich eggs. In *I. naranyae*, all adult female wasps eclosed with no mature and immature eggs (Photo, 1a), and started to produce eggs after eclosion (Figure 7). Second, the fecundity of *I. naranyae* was low (Ueno 1998). Third, it has a relatively long life span; female longevity was around one and half months at 20°C (Ueno and Tanaka 1994). In addition, the present study showed the presence of a preoviposition period in *I. naranyae*; oviposition was low in the earliest stage of female life and subsequently increased with increasing female age (Figure 1). *I. naranyae* is a host feeder.

The reproductive behavior in *I. naranyae* strongly depended on female age (Figure 1). Until 7th day after female eclosion, all test females attacked and oviposited in hosts. The behavioral pattern is well consistent with the change in the number of mature eggs in the ovary (Figure 7), indicating a close link between reproductive behavior and ovarian development.

Egg maturation patterns are highly diverse among parasitoid species, ranging from pro-ovigenic through weakly synovigenic to extremely synovigenic (Jervis et al. 2001). Because the female carries no mature eggs at eclosion, the ovigeny index (see Jervis et al. 2001) for *I. naranyae* is o; thus, this parasitoid is placed as ‘extremely’ synovigenic. After eclosion, egg loads of *I. naranyae* quickly increased even when hosts had not been provided (Figure 7). Female *I. naranyae* is therefore able to produce mature eggs from nutritional reserves that are acquired during the larval stage. This in turn enables the female to oviposit in the first several hosts encountered during the early stage of her life. As a result, oviposition precedes host-feeding in *I. naranyae* (Figure 3).

Although *I. naranyae* is a synovigenic species, which typically exhibits the five life history traits mentioned above, there are a number of synovigenic parasitoids that lack such traits. For example, an ichneumonid, *Venturia canescens*, is a non-host-feeding species, which produces a large number of small, yolkless eggs. This parasitoid, however, is highly synovigenic (Harvey et al. 2001; Eliopoulos et al. 2003). Likewise, the ovigeny index of the ichneumonid *Bathyplectes anurus* is classified as o but it is a non-host feeder and can produce many small eggs (Dowell and Horn 1977). These two parasitoids are koinobionts, in which hosts are allowed to continue the development after parasitism. Because eggs of such koinobionts can absorb nutrients required for embryogenesis from the host, females invest only a small amount of resource to each egg, and production of many eggs is thereby possible without host-feeding. Thus, the strategy of egg production in parasitoids is highly diverse, and correlations among life history traits may or may not arise depending on development mode (i.e. koinobiont versus idiobiont), availability or abundance of hosts, the size and nutritional quality of hosts, etc (e.g. Jervis et al. 2001).

### Importance of host-feeding in *I. naranyae* reproduction

The present study has shown the significance of host-feeding in female reproduction in *I. naranyae*. First, a highly positive correlation was detected between the number of oviposition events and host-feeding (Figure 6). The result shows a close link between oviposition and host-feeding activities in *I. naranyae*. Second, females that had fed on a host 3 days previously carried a greater number of mature eggs than non-fed controls (Figure 8). These results demonstrate that host-feeding facilitates egg production.

In synovigenic parasitoids, adult nutrition is known to influence production of eggs (e.g. Morales-Ramos et al. 1996; Heimpel et al. 1997; Ueno 1999; Harvey et al. 2001). Host-feeding is accepted as a major means of obtaining proteins for egg production, particularly for synovigenic species that produce relatively large, yolk-rich eggs (Jervis and Kidd 1986; Heimpel and Collier 1996; Thompson 1999). This also appears to be the case for *I. naranyae*.

Although host-feeding is an important means of obtaining nutrients for egg production in *I. naranyae*, it also incurs a reproductive cost by host-feeding. Behavioral observations have shown that *I. naranyae* obtain host body fluids by imbibing the fluids exuding after repeated ovipositor drillings. This is typically ‘destructive’ host-feeding (Jervis and Kidd 1986); in fact, hosts are heavily damaged and become unsuitable for oviposition (Ueno 1998). As a result, females lose some potential oviposition sites (i.e. host) by feeding on them. However, host-feeding allowed females to produce additional eggs (Figure 8). Thus, by feeding on a host, females lose the current opportunity for oviposition but gain resources to be used for future reproduction.

### Host-feeding and reproduction in early stages of female life

The present study has focused on host-feeding and reproduction in early stages of female life in *I. naranyae*. To date, little is known about parasitoid host-feeding in the earliest stage of female lifetime. However, investigation of this would provide some important insights into reproductive strategies of parasitoids.

Female *I. naranyae* can produce at least 30 eggs without host-feeding using nutrients stored during the larval stage (Ueno and Ueno, personal observations). It is therefore likely that females use the first 20 hosts encountered during the early stage of their life exclusively for oviposition purposes. Nevertheless, evidence was found that the majority of females used some hosts for feeding purposes in the early stage of their lifetime during which they laid relatively few eggs. Host-feeding causes direct mortality of hosts because the hosts are repeatedly attacked with the ovipositor, and hosts used for host-feeding are hence unsuitable for oviposition in *I. naranyae* (Ueno 1998). It might be difficult to adaptively explain why females use some hosts for host-feeding before they spend all nutritional reserves carried over from the larval stage. However, the time delay between host-feeding events and egg maturation could be an explanation (Collier i995b). Our study has demonstrated that it takes at least 3 days to convert ingested host materials to mature eggs (Figure 8). The presence of the time delay indicates that females have to host feed at least 3 days earlier before they will affect egg development. Host-feeding after egg exhaustion should result in 3 days loss of time during which females cannot search for hosts to oviposit in because no mature eggs are available. Thus, the timing of host-feeding could be influenced by the presence of the egg maturation delay. To our knowledge, few studies have examined the time required for converting host-feeding meal to mature eggs. However, this knowledge is essential to understanding the optimal timing of host-feeding during female lifetime (Collier 1995b).

The alternative explanation is that female *I. naranyae* may feed on hosts to gain materials not only for egg production but also for maintenance and energy for activity. If host-feeding provides the female with an essential means of gaining nutrients for maintaining its life, it would continuously use a proportion of hosts for feeding purposes throughout her life (see Jervis and Kidd 1986; Chan and Godfray 1993). Host-feeding can enhance parasitoid longevity at least in several species (Heimpel et al. 1997; Lauziere et al. 2000; Perez-Rachaud and Hardy 2001; Giron et al. 2002) but not for others (Morales-Ramos et al. 1996; Heimpel et al. 1997; Ueno 1999a; Zaviezo and Mills 1999). It is not known whether host-feeding plays a role in enhancing female longevity of *I. naranyae*.

In any case, female *I. naranyae* appear to accumulate nutrients during the early phase of her life. Recent studies have shown that other female parasitoids can allocate to reproduction nutrients gained early in female life (Rivero and Casas 1999; Fadamiro and Heimpel 2001; Rivero et al. 2001). Although it is not known whether *I. naranyae* can store nutrients gained via host-feeding over a relatively long time to produce eggs in later stages of the life, this is likely to be the case for *I. naranyae*.

Curiously, considerable variation was observed for the timing of females to begin oviposition and host-feeding (Figure 2). A positive relationship between the timing of the first oviposition and host-feeding suggests that females that started oviposition earlier started to host feed earlier. However, this relationship explains only a 20% of the variation in the timing of host-feeding (Figure 2). Other unknown factors are thus involved. A possible factor is physiological state of the ovipositing female. It is known that female parasitoids are more likely to feed on hosts when egg loads are smaller (Collier et al. 1994; Heimpel *et al*. 1995; Ueno 1999b). The difference in egg load among females may determine the timing of the first host-feeding during their lifetime.
